# Novel DNA methylation biomarkers show high sensitivity and specificity for blood-based detection of colorectal cancer—a clinical biomarker discovery and validation study

**DOI:** 10.1186/s13148-019-0757-3

**Published:** 2019-11-14

**Authors:** Sarah Østrup Jensen, Nadia Øgaard, Mai-Britt Worm Ørntoft, Mads Heilskov Rasmussen, Jesper Bertram Bramsen, Helle Kristensen, Peter Mouritzen, Mogens Rørbæk Madsen, Anders Husted Madsen, Kåre Gotschalck Sunesen, Lene Hjerrild Iversen, Søren Laurberg, Ib Jarle Christensen, Hans Jørgen Nielsen, Claus Lindbjerg Andersen

**Affiliations:** 1 Department of Molecular Medicine, Aarhus University Hospital, Palle Juul-Jensens Boulevard 99, 8200 Aarhus N, Denmark; 2 Exiqon, Vedbæk, Denmark; 3 Herning Regional Hospital, Herning, Denmark; 4 Randers Regional Hospital, Randers, Denmark; 5 Department of Surgery, Aarhus University Hospital, Aarhus, Denmark; 6 Center for Surgical Research, Department of Surgical Gastroenterology, Hvidovre Hospital, Hvidovre, Denmark; 7 Institute of Clinical Medicine, University of Copenhagen, Copenhagen, Denmark

**Keywords:** DNA methylation, Epigenetic biomarkers, Cancer, Colorectal cancer, Liquid biopsy, Circulating tumour DNA, Early detection

## Abstract

**Background:**

Early detection plays an essential role to reduce colorectal cancer (CRC) mortality. While current screening methods suffer from poor compliance, liquid biopsy-based strategies for cancer detection is rapidly gaining promise. Here, we describe the development of TriMeth, a minimal-invasive blood-based test for detection of early-stage colorectal cancer. The test is based on assessment of three tumour-specific DNA methylation markers in circulating cell-free DNA.

**Results:**

A thorough multi-step biomarker discovery study based on DNA methylation profiles of more than 5000 tumours and blood cell populations identified CRC-specific DNA methylation markers. The DNA methylation patterns of biomarker candidates were validated by bisulfite sequencing and methylation-specific droplet digital PCR in CRC tumour tissue and peripheral blood leucocytes. The three best performing markers were first applied to plasma from 113 primarily early-stage CRC patients and 87 age- and gender-matched colonoscopy-verified controls. Based on this, the test scoring algorithm was locked, and then TriMeth was validated in an independent cohort comprising 143 CRC patients and 91 controls. Three DNA methylation markers, *C9orf50*, *KCNQ5,* and *CLIP4*, were identified, each capable of discriminating plasma from colorectal cancer patients and healthy individuals (areas under the curve 0.86, 0.91, and 0.88). When combined in the TriMeth test, an average sensitivity of 85% (218/256) was observed (stage I: 80% (33/41), stage II: 85% (121/143), stage III: 89% (49/55), and stage IV: 88% (15/17)) at 99% (176/178) specificity in two independent plasma cohorts.

**Conclusion:**

TriMeth enables detection of early-stage colorectal cancer with high sensitivity and specificity. The reported results underline the potential utility of DNA methylation-based detection of circulating tumour DNA in the clinical management of colorectal cancer.

## Introduction

Colorectal cancer (CRC) claims more than 880,000 lives each year worldwide and is a major public health concern in the Western world [1, 2]. Much of the morbidity and mortality of CRC result from diagnosis at late stages, where the therapeutic intervention is less effective. CRC screening using faecal occult blood testing and bowel endoscopy has been shown to enable early detection and reduce CRC mortality [3, 4]. However, the compliance rates in CRC screening, based on either direct endoscopy or testing for occult blood in faeces and subsequent colonoscopy, are poor to modest [5]. A recent study evaluated the sample preference, blood or faeces, for a CRC screening test among screening-aged individuals and found that 78% of the survey participants preferred to provide a blood sample [6]. Hence, blood-based tests could potentially improve compliance in population-based screening programmes, given their minimally invasive nature and straightforward implementation in routine medical examinations [7]. Blood contains numerous analytes, including circulating cell-free (cfDNA). We and others have previously shown that in individuals with cancer, some of the cfDNA may originate from the tumour (circulating tumour DNA, ctDNA) [8–11]. Thus, cfDNA has the potential to distinguish healthy individuals from cancer patients. Recently, analyses using DNA mutation and methylation-based strategies for detection of ctDNA have suggested that such approaches may provide new avenues for early cancer diagnosis [12–14]. While both strategies have shown promises, mutation-based strategies are particularly challenged by the limited number of recurrent mutations available to distinguish tumour and normal cfDNA in a cost-efficient manner. By contrast, tumour-specific DNA hypermethylation occurs early in tumour development and is highly recurrent [15]. Consistently, several promising DNA methylation markers have been reported [16–18], though none have yet shown sufficient clinical performance to be considered implemented in CRC screening [19]. Here, we report the results of a combined discovery and validation study, aimed at identifying novel blood-based DNA methylation markers, and document their ability to efficiently discriminate healthy individuals from patients with early-stage CRC.

## Results

**Fig. 1:**
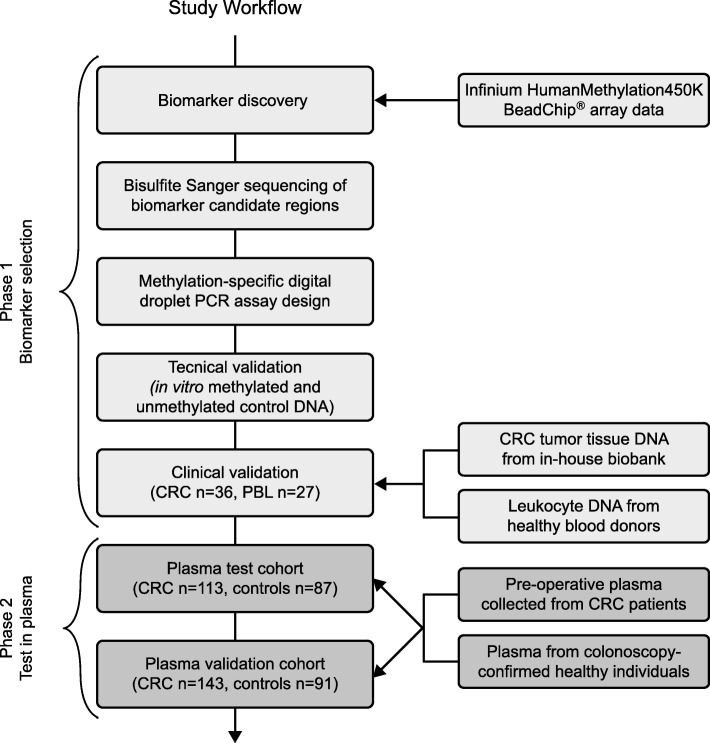
Overview of study workflow. Infinium Human Methylation450K BeadChip® array data from > 4000 samples including CRC, PBL, normal colorectal mucosa, and other cancer types were used to identify CRC-specific DNA methylation biomarkers. The methylation pattern of candidate marker regions was confirmed by bisulfite Sanger sequencing of paired CRC tissue, normal colorectal mucosa, and PBLs. Methylation-specific ddPCR assays targeting candidate regions were designed and optimized, and clinical validation was performed by evaluating assays in CRC tumour tissue (*n* = 36) and PBL from blood donors (*n* = 27). The top three candidates were analysed in two independent cohorts consisting of plasma from CRC patients and controls. CRC colorectal cancer, PBLs peripheral blood leucocytes, ddPCR droplet digital PCR

The study was conducted in two phases. In phase 1, marker discovery was performed including DNA methylation marker selection, methylation-specific droplet digital PCR (ddPCR) assay design, and testing in clinical tissue samples. In phase 2, the selected markers were applied to two independent plasma cohorts from CRC patients and matched controls (Table 1). An overview of the study workflow is presented in Fig. 1.

**Table 1:** Patient characteristics and demographics of plasma cohorts

	Test cohort		Validation cohort	
	CRC	Controls	CRC	Controls
Total (*n*)	113	87	143	91
Sex *n* (%)				
Female	56 (49.6)	39 (44.8)	62 (43.0)	46 (50.5)
Male	57 (50.4)	48 (55.2)	81 (57.0)	45 (49.5)
Age (years)				
mean (SD)	70.8 (9.1)	67.2 (6.6)	73.2 (8.9)	66.6 (5.6)
UICC stage, *n* (%)				
Stage I	16 (14)	-	25 (18)	-
Stage II	68 (60)	-	75 (52)	-
Stage III	22 (20)	-	33 (23)	-
Stage IV	7 (6)	-	10 (7)	-
Tumour diameter (mm)				
Mean (SD)	53.3 (26.5)	-	53.3 (32.1)	-
Histological type *n* (%)				
Adenocarcinoma	106 (93.8)	-	134 (93.7)	-
Mucinous adenocarcinoma	4 (3.5)	-	7 (4.9)	-
Signet ring cell adenocarcinoma	1 (0.9)	-	2 (1.4)	-
Unspecified or missing	2 (1.8)	-	0 (0)	-
Localization *n* (%)				
Right (cecum, ascending, transverse)	48 (42.5)	-	79 (55.2)	-
Left (descending, sigmoid)	47 (41.6)	-	54 (37.8)	-
Rectum	18 (15.9)	-	10 (7.0)	-

*CRC* colorectal cancer, *UICC* Union of International Cancer Control, *SD* standard deviation

### DNA methylation biomarker discovery and validation of candidate marker regions by bisulfite sequencing

**Fig. 2:**
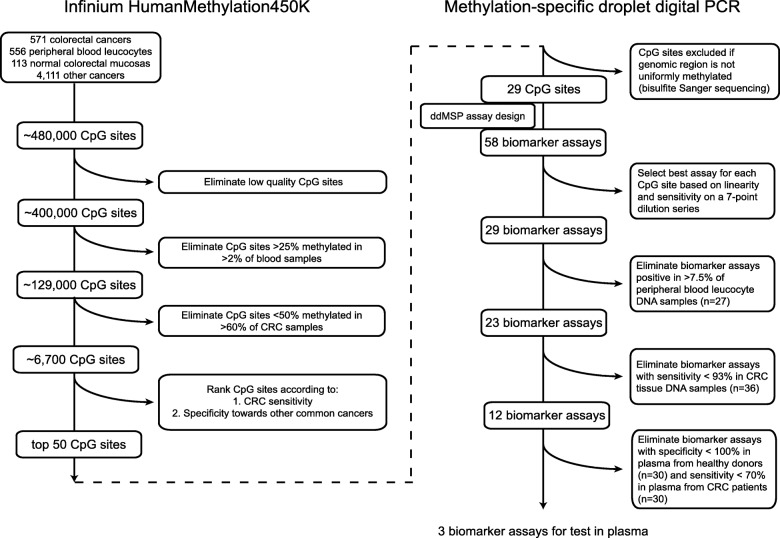
Schematic representation of biomarker discovery and validation pipeline. Infinium HumanMethylation450K Beadchip® array data were used to evaluate the methylation status of CpG sites in CRC, PBL, normal mucosa, and other cancer types (left panel). We excluded CpG sites that were methylated in blood cells and CpG sites with low methylation in CRC. The remaining 6700 CpG sites were ranked according to CRC sensitivity and specificity against other common cancers. To confirm uniform methylation in genomic regions of candidate CpG sites, bisulfite Sanger sequencing was performed on paired samples of CRC, PBL, and normal colorectal mucosa. Twenty-nine of the top 50 CpG sites were located in regions compatible with successful methylation-specific ddPCR assay design. A total of 58 methylation-specific ddPCR assays were designed for the 29 CpG sites (markers) and tested in a sequence of validation steps (right panel). Assays were excluded if their performance was suboptimal in methylated and unmethylated control DNA, PBLs, and CRC tissue or in plasma from CRC patients. Three markers passed all selection criteria. CRC colorectal cancer, PBL peripheral blood leukocytes, ddPCR droplet digital PCR

Biomarker discovery was performed using a combination of in-house-produced and publicly available Infinium HumanMethylation450K BeadChip® (450K) DNA methylation array data from 571 CRC tumours, 113 adjacent normal colorectal mucosa samples, 556 blood cell populations, and 4111 tumour samples from 17 different cancer types, including major cancer types such as breast, prostate, and lung cancer. A stepwise strategy was applied to identify and select CRC-specific DNA methylation marker candidates (Fig. 2). CpG sites hypermethylated in CRCs, unmethylated in peripheral blood leukocytes (PBLs), and minimally methylated in other cancers and normal colorectal mucosa were prioritized. However, methylation status in normal mucosa was not deemed critical, as DNA from intestinal cells is not found in the circulation of healthy individuals [20]. The 50 top-ranked CpG sites were selected for further analysis. While it is straightforward to design methylation-specific assays targeting uniformly methylated genomic regions, this is not the case for heterogeneously methylated regions. Thus, to confirm uniform and CRC-specific methylation in candidate regions, we performed bisulfite sequencing of 2 to 7 sets of matched blood, tumour, and normal mucosa (Additional file 1: Figure S1, Additional file 9: Table S2). This identified 29 uniformly methylated candidates, which were selected for assay design (Fig. 2).

### Biomarker candidate assay development and technical validation

An average of two methylation-specific ddPCR assays, targeting bisulfite-converted DNA, were designed, tested, and optimized for the 29 CpG candidate sites, 58 assays in total (Additional file 7). The validation tests and the order in which they were performed are described in Fig. 2. The technical sensitivity of the assays was evaluated using a 7-point dilution series, where 0, 8, 16, 32, 64, 128, and 256 methylated DNA copies were mixed with 20,000 human unmethylated DNA copies (data not shown). The best performing assay, based on linearity and sensitivity, was selected for each candidate CpG site, leaving one assay per CpG site (Additional file 9: Table S3). All selected assays were able to detect 8 copies of methylated DNA in a background of 20,000 copies of unmethylated DNA. None of the assays amplified unmethylated DNA.

### Biological validation of biomarker candidates in clinical tissue and plasma samples

**Fig. 3:**
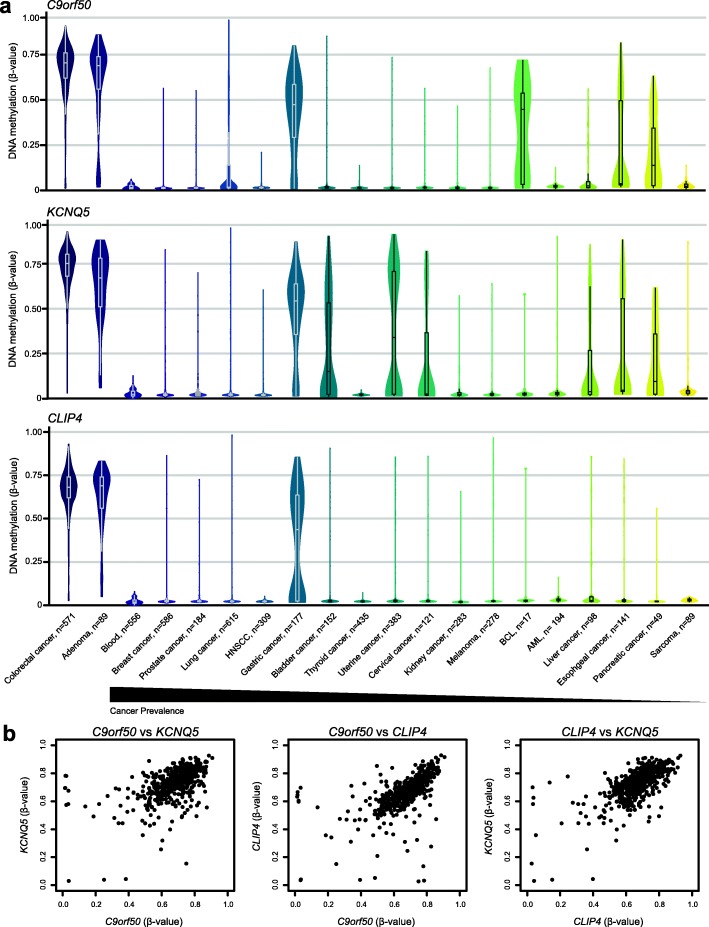
DNA methylation of *C9orf50*, *KCNQ5*, and *CLIP4*. **a** DNA methylation levels (Infinium HumanMethylation450K BeadChip® array data) of the three markers *C9orf50*, *KCNQ5*, and *CLIP4* in 571 individual CRC tumours, 556 PBL samples, and 4111 samples from other cancer types. Each of the three markers are hypermethylated (*β*-value > 0.35) in > 97% of CRC tumours. **b** Correlation of DNA methylation levels in CRC tissue of the *C9orf50*, *KCNQ5*, and *CLIP4* markers. HNSCC head and neck squamous cell carcinoma, BCL B-Cell lymphoma, AML acute myeloid leukaemia

To assess specificity, the 29 assays were applied to PBLs from 27 healthy individuals. The majority showed no signal in these samples (Additional file 2: Figure S2a), but six markers showed signal in more than 7.5% of the PBL samples and were excluded from further analyses. To assess the clinical sensitivity, i.e. the proportion of tumours testing positive with a given assay, the remaining 23 assays were applied to DNA from 36 early-stage (Union of International Cancer Control (UICC) stage I-II) CRC tumours (Additional file 2: Figure S2b). Twelve assays that detected methylated DNA fragments in more than 93% of tumours and showed no signal in the PBLs were selected and further tested in plasma samples from 30 CRC patients and 30 controls with no neoplasia detected at colonoscopy (colonoscopy-negative). The three best performing assays, detecting methylated DNA fragments in more than 70% of the CRC patient plasma samples and in none of the control samples (100% specificity) (Additional file 3: Figure S3), were selected as the final DNA methylation marker panel. They target CpG sites located in the 5′-regions of the genes *C9orf50*, *KCNQ5*, and *CLIP4* (Additional file 4: Figure S4) that were found to be concurrently hypermethylated in CRC and adenoma tissues, but unmethylated in blood cells and in most other cancer types (Fig. 3, Additional file 9: Table S1). Furthermore, 100% of CRC tumours showed hypermethylation of at least one of the three markers.

### Biomarker evaluation in plasma from early-stage CRC patients and matched controls

**Fig. 4:**
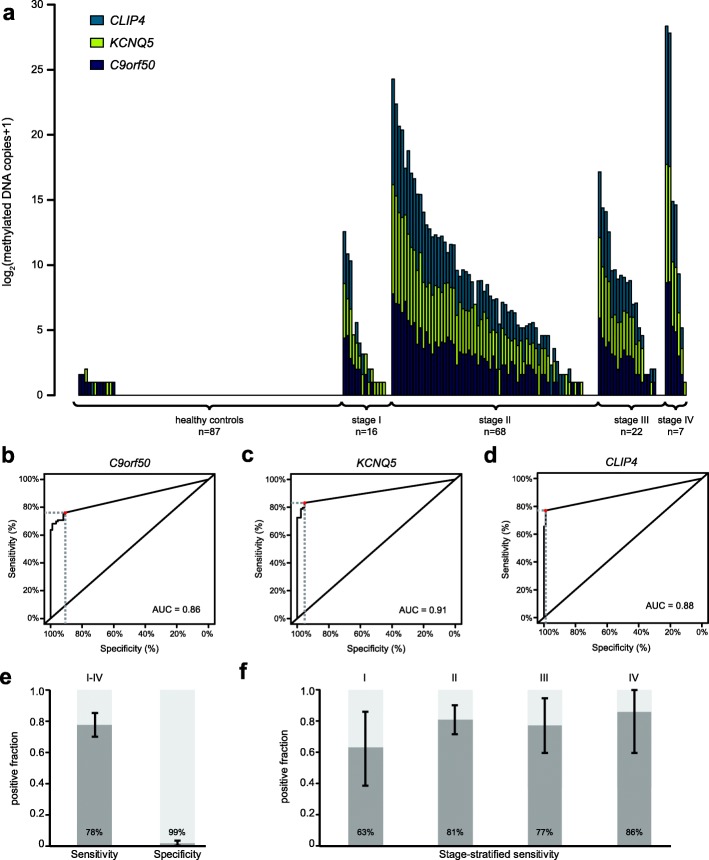
Detection of *C9orf50*, *KCNQ5*, and *CLIP4* markers in plasma (test cohort). **a** Methylation-specific ddPCR was performed on 4500 copies of bisulfite-converted cfDNA to detect methylated *C9orf50*, *KCNQ5*, and *CLIP4* in plasma samples from CRC patients and controls in the test cohort. The total number of methylated DNA copies (sum of the three markers) are recorded on the *y*-axis and CRC samples are arranged by UICC stage. **b**–**d** ROC curves (right) from test of *C9orf50*, *KCNQ5*, and *CLIP4* individual markers in plasma. The red dots and dashed lines indicate the sensitivity and specificity when calling samples positive if they contained any methylated DNA. **e** Sensitivity and specificity by the TriMeth test in plasma. **f** UICC stage-stratified sensitivity of TriMeth in plasm. Error bars represent 95% CI. ddPCR droplet digital PCR, CRC colorectal cancer, UICC Union for International Cancer Control, ROC receiver operating characteristics

To evaluate the performance in plasma, we applied the three markers to a test cohort of plasma samples from 113 CRC patients and 87 age- and gender-matched, colonoscopy-negative controls from the Danish national CRC screening programme (Table 1). The controls had a positive faecal immunochemical test (FIT), but the subsequent colonoscopy showed no findings of CRC or adenoma. The three marker assays and a cytosine-free (CF) control assay, that quantifies DNA regardless of CpG methylation status and bisulfite treatment, were applied to each sample. The bisulfite-converted cfDNA (BS-cfDNA) was quantified using ddPCR (copies per millilitre plasma; range, 210–23,798; median 968). The mean recovery after bisulfite conversion was 36.4% (95% CI 35.1–37.7). For each ddPCR reaction, a fixed input of 4500 BS-cfDNA copies was used. This input level is theoretically sufficient for detection of methylated DNA in individuals where the ctDNA fraction constitute down to 0.02% (1/4500 × 100) of the total cfDNA. To minimize the BS-cfDNA needed to measure the markers, we interrogated if it was efficient to run them and the CF control assay, in duplex reactions. All pair-wise combinations were tested in terms of fluorescence intensity of the positive droplets and the technical sensitivity compared to singleplex reactions. Based on this, we chose to duplex *C9orf50* with *KCNQ5,* and *CLIP4* with the CF control assay (Additional file 5: Figure S5, Additional file 9: Tables S3 and S5). The number of methylated DNA molecules detected in plasma from the 113 CRC patients and 87 controls when using the duplex reactions are shown in Fig. 4a. For controls, only few samples showed signs of methylated DNA and typically only one marker was positive. In contrast, the majority of cases were positive, generally with more than one marker and a higher number of methylated copies than controls (Fig. 4a). Receiver operating characteristics (ROC) curves illustrate the ability of the individual markers *C9orf50* (area under the curve (AUC) = 0.86), *KCNQ5* (AUC = 0.91) and *CLIP4* (AUC = 0.88) to discriminate CRC patients from controls (Fig. 4b–d, Additional file 6: Figure S6). The sensitivities were 76% for *C9orf50*, 83% for *KCNQ5* and 77% for *CLIP4*, when calling samples positive if they contained any methylated DNA (Fig. 4b–d). The corresponding specificities were 91%, 95% and 99%. Due to their high specificity for CRC, the likelihood of more than one marker being false positive in the same individual is low. Therefore, we investigated if specificity was improved by combining the markers using a simple two-of-three algorithm, and in parallel, we assessed if it affected sensitivity, especially for early-stage CRC. The combined test was termed “TriMeth”, and as indicated samples were scored positive if two or more markers were positive (the individual markers were called positive if they detected any methylated DNA). The sensitivity of TriMeth was 78% and the specificity was 99% (Fig. 4e). The stage-stratified sensitivity was 63%, 81%, 77%, and 86% for stage I, II, III and IV, respectively (Fig. 4f). Additional file 9: Table S4 shows the stage-stratified sensitivities and specificities for individual markers.

### Independent validation of TriMeth

**Fig. 5:**
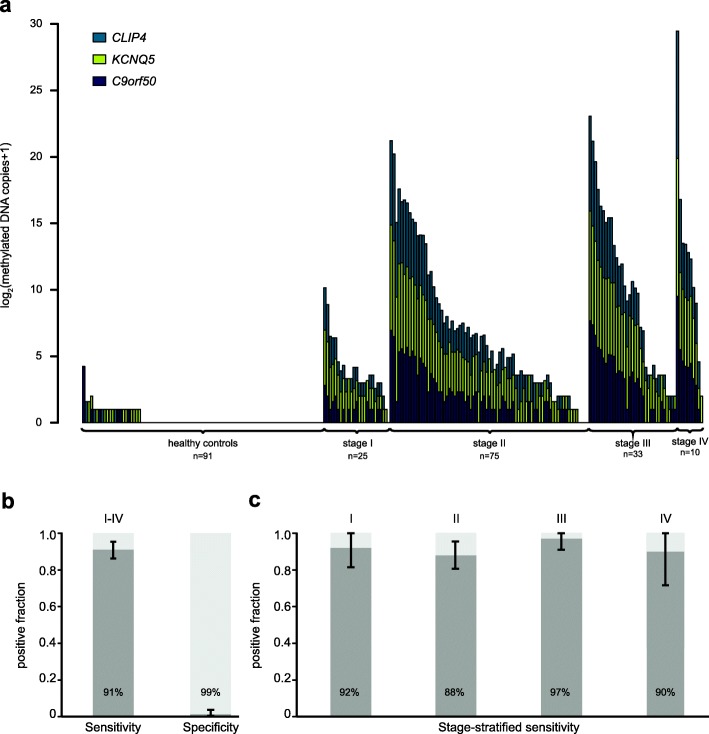
Independent validation of TriMeth in plasma. **a** Methylation-specific ddPCR was performed on cfDNA purified from plasma of CRC patients and controls (validation cohort) to detect methylated *C9orf50*, *KCNQ5*, and *CLIP4*. **b** Sensitivity and specificity of the TriMeth tes. **c** UICC stage-stratified sensitivity of TriMeth. Error bars represent 95% CI. ddPCR droplet digital PCR, CRC colorectal cancer, UICC Union for International Cancer Control

To evaluate whether these results could be replicated in an independent cohort, the two-of-three scoring algorithm was locked, and TriMeth applied to a validation cohort consisting of plasma samples from 143 stage I-IV CRC patients and 91 colonoscopy-negative controls from the Danish national CRC cancer screening programme (Table 1). In clinical practice, it is impractical to use equal amounts of cfDNA input, which effectively means that the needed plasma volume varies. The critical factor is to analyse sufficient cfDNA to justify calling a sample negative for methylated DNA. From the test cohort, we found that the 20th percentile for cfDNA per millilitre plasma was 625 copies. Thus, by using 16 ml of plasma, we expected at least 80% of the samples to have more than 5000 copies of BS-cfDNA input per ddPCR duplex. Therefore, for the validation cohort a plasma volume of 16 ml was used. After bisulfite conversion, the BS-cfDNA was quantified by ddPCR and split into two ddPCR reactions. Results showed that more than 90% of the samples had an input of minimum 5000 copies per ddPCR reaction (range 2244–143,880, median 7612). Recovery after bisulfite conversion was 54.1% (95% CI 52.8–55.3). The number of methylated DNA molecules detected in plasma from CRC patients and controls in the validation cohort is shown in Fig. 5a. The TriMeth test had a sensitivity of 91% and a specificity of 99% in this cohort (Fig. 5b). The UICC stage-stratified sensitivity was 92%, 88%, 97%, and 90% for stage I, II, III, and IV, respectively (Fig. 5c).

### Quantities of methylated DNA fragments in cases and controls

The basis of the present CRC detection approach is that the methylated DNA templates detected in plasma are derived from dying cancer cells. In agreement, methylated DNA was rarely detected in cfDNA from controls (Figs. 4 and 5). Only 1% (2/178) of the controls were positive and most often by one marker only. Moreover, these positive control samples contained a median of only 0.1 (95% CI 0.1–0.2) methylated DNA fragments/ml plasma. By contrast, methylated DNA was detected, by two or more markers, in 85% (218/256) of samples from CRC patients, with a median of 1.3 (95% CI 0.9–2.0) methylated fragments/ml plasma.

## Discussion

Early detection is key to increase eligibility for curative intervention and thereby reduce CRC mortality. CRC screening has proven efficient for early detection of cancerous lesions, but current screening strategies suffer from low compliance rates. Here, we report the identification of three CRC-specific DNA methylation markers *C9orf50*, *KCNQ5*, and *CLIP4* and demonstrate their utility (the TriMeth test) for detection of CRC-specific ctDNA in human blood samples. TriMeth was applied to plasma from two independent cohorts and on average detected ctDNA in 85% (218/256) of CRC patients. At this sensitivity, the specificity was 99% in both cohorts, only 2 of 178 controls scored positive. We cannot be certain that the two “false positive” individuals did not have a CRC that was missed by the colonoscopy, but classifying them as false positives provides the most conservative approach to interpretation of the data. One of the most important attributes of a screening test is the ability to detect early-stage cancers. In the present study, the TriMeth test achieved high sensitivity for all stages, with average sensitivities of 80% for stage I, 85% for stage II, 89% for stage III, and 88% for stage IV. While plasma from patients with colorectal adenomas, the benign counterpart to adenocarcinomas, were not investigated in this study, our analyses of adenoma tumour tissues revealed the same TriMeth signals as found in colorectal cancer tissues (Fig. 3a). Hence, if adenomas shed tumour DNA to the circulation then they can potentially be detected. In the future, TriMeth-based identification and removal of adenomas may be a path to reduce CRC incidence.

In the marker discovery phase of the present study, extensive efforts were made to ensure that the selected DNA methylation markers were specifically hypermethylated in CRC compared to other cancer types and normal cells of haematopoietic origin. However, we cannot exclude that other normal tissues might have a DNA methylation profile similar to that observed in CRC. One can imagine that such tissues, in some situations (e.g. disease-related), may shed DNA into the circulation and cause single markers to become positive, similar to what we observed. To overcome this issue, we included three synchronously methylated CRC markers in the TriMeth test. We hypothesized that while normal tissues might have a DNA methylation pattern similar to CRC at a single site, it was unlikely to happen at multiple sites. Consequently, we expected to be able to discriminate methylated DNA released from cancer cells and other cells, by requiring at least two out of three markers to be methylated, and indeed this was confirmed. TriMeth showed a specificity of 99%. The sensitivity and, particularly the specificity, of TriMeth are favourable to that of the frequently used FIT test. In a recent meta-analysis, FIT was reported to have a sensitivity of 71% (95% CI 58–81%) and a specificity of 94% (95% CI 91–96%) when the reference standard was colonoscopy, as in the present study [21]. This suggests that TriMeth may have potential as a screening test. As TriMeth is blood-based, a patient compliance higher than for FIT may be expected [6], which could potentially lead to detection of a larger proportion of the CRCs in the screening population. TriMeth could potentially also be used to supplement existing FIT-based CRC screening programmes, e.g. as an option for the invitees that refuse the FIT test, or for triaging FIT-positives to colonoscopy to reduce the number of colonoscopies needed to detect one CRC [22]. Potentially, TriMeth could also be used in a postoperative setting, to identify patients with minimal residual disease and relapse. We observed significant inter-patient variation in the cfDNA quantity per millilitre plasma. To minimize the risk of falsely classifying a sample as negative due to insufficient cfDNA input, equal quantities of cfDNA input were used in the test cohort. However, analysing equal amounts of cfDNA is not practically feasible. Hence, 16 ml of plasma was used in the validation cohort, which ensured that a minimum of 5000 cfDNA copies were analysed per ddPCR for > 90% of samples. This volume of plasma exceeds what previous studies have used, but it is indeed feasible in a clinical setting [23]. However, the ability to detect very early-stage tumours and adenomas will ultimately be limited by the level of tumour DNA shedding and the presence of ctDNA fragments in the collected blood volume. It may very well be that small and early-stage tumours, which are shedding only limited amounts of DNA to the circulation, may be undetectable at standardly collected blood volumes. If the number of tumour DNA fragments in the available blood volume falls below the detection threshold of the used methods, the lesion will go undetected. A few limitations of the study should be acknowledged. Firstly, the CRC patients were individuals with known cancers, most of which were diagnosed on the basis of symptoms. The fraction of stage I tumours will probably be higher among asymptomatic, screened individuals, and consequently the sensitivity of detection in a screening population might be less than reported here. To limit this bias, the inclusion was focused on CRC patients with early-stage disease to mimic a true screening setting. Secondly, while the controls were recruited among asymptomatic individuals participating in the Danish national CRC screening programme, they were selected among the FIT-positive and colonoscopy-negative subset. Consequently, they may not reflect the screening population in all details. Thirdly, because our markers show weak to moderate DNA methylation signals in other gastrointestinal cancers, particular gastric cancer (Fig. 3a), there is a risk that TriMeth might become positive in a fraction of non-CRC gastrointestinal cancer patients. Consequently, in a clinical setting it will be important to consider the clinical follow-up procedure after a positive TriMeth test. For instance, if the follow-up colonoscopy is negative, it may be advisable to do a gastroscopy.

## Conclusion

In summary, we have developed and validated a sensitive and specific DNA methylation marker TriMeth test for the detection of ctDNA released by CRCs. TriMeth awaits validation in an asymptomatic setting, but the findings reported here emphasize the potential utility of our DNA methylation markers as a basis for minimally invasive, blood-based, sensitive, and specific early tumour detection for cancer interception.

## Methods

### Study design

This study presents a multi-phased marker discovery and validation study with retrospective analysis of cfDNA using Locked Nucleic Acid™-enhanced methylation-specific ddPCR assays, to detect CRC-specific DNA methylation alterations in plasma from CRC patients and controls. We analysed plasma from 434 individuals, including 178 controls (FIT-positive and colonoscopy-negative) and 256 stage I-IV CRC patients with most patients exhibiting early-stage disease (Table 1). The sensitivity and specificity of three DNA methylation markers were evaluated in two independent plasma cohorts. No statistical methods were used to predetermine sample size.

### Patient samples

Between May 2014 and December 2014, pre-operative plasma was collected from 256 patients diagnosed with stage I-IV CRC at the Surgical Departments of Aarhus University Hospital and the Regional Hospitals in Randers and Herning. In the same period, control plasma was collected from 178 age- and gender-matched FIT-positive participants with colonoscopy-verified clean colons, no previous cancer diagnosis, and no comorbidities except for hypertension in the Danish colorectal cancer screening programme [23]. The samples were organized in two cohorts and general demographic information is presented in Table 1. Informed consent was obtained from all participating patients and controls. Tumour samples from CRC patients were collected at the Surgical Departments of Aarhus University Hospital, Randers Regional Hospital and Herning Hospital in Denmark. The tissue was snap-frozen in liquid nitrogen within 30 min from end of tumour resection and stored at − 80 °C. PBLs were isolated from 10 ml of blood collected from presumed healthy Danish blood donors at the Blood Bank, Aarhus University Hospital, Denmark.

### Biomarker discovery and filter criteria

For the marker discovery, we used DNA methylation datasets generated by 450K arrays, which quantifies the DNA methylation levels of 482,421 CpG loci by calculating the ratio (*β*-value) of intensities between methylated and unmethylated alleles. The 450K data was either available in-house [24, 25] or through “Marmal-aid”, a public database for Infinium HumanMethylation450 datasets [26]. The public data were primarily generated by “The Cancer Genome Atlas” project (https://portal.gdc.cancer.gov/). All datasets were processed (*β*-value calling and normalization) using standard settings by the ChAMP R-package [27]. CRC-specific marker candidate CpG sites were identified using the filter steps shown in Fig. 2, and 50 CpG sites were selected for assay design and further evaluation.

### Bisulfite sequencing

Primers flanking selected candidate CpG sites were designed using Bisearch [28]. The primers were placed 100–250 nucleotides upstream and downstream of the index CpG site, to ensure that the amplicon covered several CpG sites. In order to amplify the candidate regions of interest, ranging from ~ 200–500 nucleotides, PCR reaction mixes were prepared containing 1.5 μl Tempase Key Buffer (Amplicon), 0.2 μl Tempase Hot Start DNA Polymerase (Amplicon), 1.5 μl dNTP mix (1.25 mM) (Roche), 0.5 forward primer (10 μM), 0.5 reverse primer (10 μM), and 9.8 μl AccuGENE™ Molecular Biology Water (Lonza). One microlitre bisulfite-converted template DNA from colorectal tumours, PBLs, or normal colorectal mucosa was added to a final volume of 15 μl and PCR reactions were run on a C1000 thermal cycler (Bio-Rad Laboratories). Primers are listed in Additional file 9: Table S2. Following PCR amplification, 5 μl of each amplicon were visualized by agarose gel electrophoresis in Tris-acetate-EDTA (TAE) buffer (Fagron) with ethidium bromide (EtBr) to test for correct band size. GeneRuler™ 100 bp Plus DNA Ladder (Thermo Scientific) was used as a molecular marker. To remove excess primers and dNTPs, 2 μl PCR products were treated with 1 μl FastAP Thermosensitive Alkaline Phosphatase (1 μmol/μl) (Life Technologies) and 1 μl EXO1 (20,000 U/ml) (New England Biolabs) diluted 1:4 in 10x Exonuclease I Reaction buffer (New England Biolabs). The mix was incubated on a C1000 thermal cycler (Bio-Rad) at 37 °C for 15 min and 85 °C for 15 min. Sanger sequencing was performed using 0.5 μl Ready Reaction mix (Life Technologies), 1.5 μl Big-Dye sequencing buffer (Life Technologies), 1 μl primer (2 pmol/μl), 2 μl purified PCR product, and RNase-free water to a total volume of 10 μl. The product was ethanol/EDTA/na-acetate precipitated and sequenced on a 3130x Genetic Analyser (Applied Biosystem). Results were analysed using Sequencer 5.1 software (Gene Codes Cooperation).

### Design and optimization of methylation-specific droplet digital PCR assays

Methylation-specific ddPCR primers and probes were designed to cover candidate regions validated by bisulfite sequencing and be exclusively specific for methylated, bisulfite-treated DNA. To increase assay specificity and reduce amplicon lengths, LNA™, that have an increased affinity for complementary DNA bases [29], were incorporated into primers and probes. In the assay design, the LNA™ Oligo Optimizer tool (https://www.exiqon.com/ls/Pages/ExiqonOligoOptimizerTool.aspx) was used to ensure LNA™ oligo designs with a self-complementarity and secondary structure score below 40. The annealing temperature (*T*_m_) of the LNA™-enhanced oligos was predicted using the LNA™ Oligo Tm Prediction tool (https://www.exiqon.com/ls/Pages/ExiqonTMPredictionTool.aspx). Primers were designed to have *T*_m_ = 59–61 °C and probe *T*_m_ = primer *T*m + 5–10 °C. Primers were manufactured by Qiagen and probes manufactured by LGC biosearch technology. All assays were optimized for ddPCR according to the guidelines for minimum information for publication of quantitative digital PCR experiments [30] (digital MIQE checklist shown in Additional file 9: Table S6). Primer and probe sequences and assay details are shown in Additional file 9: Table S3.

### Tissue and blood processing, including DNA isolation

DNA was extracted from fresh frozen tissue using the Gentra Puregene Tissue Kit (Qiagen) as specified by manufacturer. DNA from PBLs was purified on a QIAsymphony robot (Qiagen) using the QiaSymphony DSP DNA mini kit (Qiagen) as specified by manufacturer, eluted in 1.5 ml Eppendorf tubes (Eppendorf AG) and stored at − 80 °C until use (< 2 months). Whole blood was collected in BD Vacutainer K2 EDTA tubes (Becton Dickinson) and processed within 2 h from venipuncture. Blood from controls was collected after bowel cleansing but prior to colonoscopy, and blood from CRC patients was collected prior to surgery. Blood samples from CRC patients and controls were processed identically. To separate plasma from cellular components, plasma was double centrifuged at 3000*g* for 10 min at 20 °C and stored in cryotubes (TPP) at − 80 °C until the time of DNA extraction (< 3 years). Plasma was thawed at room temperature and cfDNA from 8 to 24 ml of plasma was extracted using a QIAsymphony robot and the QIAamp® Circulating Nucleic Acids kit (Qiagen) as specified by manufacturer. Purified cfDNA was eluted in LoBind tubes or LoBind 96-well plates (Eppendorf AG) and stored at − 80° until further use (< 2 months). Purification efficiency and analysis for contamination with DNA from lysed lymphocytes were assessed by ddPCR as previously described [10]. In brief, a fixed amount of soybean CPP1 DNA fragments was added to each plasma sample prior to extraction. Purification efficiency was calculated as the percent recovery of CPP1 fragments following cfDNA extraction (CPP1 assay). Lymphocyte DNA contamination was estimated by an assay targeting the VDJ rearranged IGH locus specific for B cells (PBC assay). The median purification efficiency was 74.4% (interquartile range 65.8–84.0%). A minor contamination with lymphocyte DNA, was observed in 6.8% of samples, but since their cfDNA levels did not deviate from the rest, these samples were flagged rather than excluded.

### Bisulfite conversion

Prior to bisulfite conversion, cfDNA was dried using vacuum centrifugation (speedVac, Concentrator plus 5350, Eppendorf AG) at 30 °C and resuspended in 20 μl AccuGENE™ Molecular Biology Water (Lonza). All DNA samples were bisulfite-converted using the EZ-96 DNA Methylation-Direct™ MagPrep kit (Zymo Research) according to manufacturer’s instructions, but with the following modifications to the volumes used: 60 μl CT conversion reagent, 280 μl M-Binding Buffer, 5 μl MagBinding Beads, 185 μl M-Wash Buffer, 93 μl M-Desulphonation Buffer, and 25 μl M-Elution Buffer. Methylated and unmethylated DNA standards (Zymo Research) were included in each bisulfite conversion batch, as positive and negative controls. Reactions were performed on a S1000 Thermal cycler (Bio-Rad). The bisulfite-converted DNA samples were analysed using ddPCR immediately after completed bisulfite conversion or stored at − 20 °C until use (< 2 months).

### DNA quantification before and after bisulfite conversion

Native DNA samples were quantified by ddPCR using assays targeting two reference regions located on chromosome 1 (CF assay) and chromosome 3 (Chr3 assay) (Additional file 9: Table S5). Both assays are located in regions that only rarely show copy number aberrations in cancer. Reported quantities are the average of the two assays. The CF assay was furthermore designed to amplify a cytosine-free region of the genome, thereby, enabling the use of the same assay for quantification of both native and bisulfite-converted DNA. The CF assay was used for DNA quantification and recovery assessments after bisulfite conversion. Recovery was calculated as the CF quantity after bisulfite conversion divided by the CF quantity before. Using the same assay before and after bisulfite treatment facilitates an unbiased recovery estimate.

### Droplet digital PCR

All reagents, except from template DNA, were prepared in an isolated pre-PCR room to avoid contamination. The reaction master mix was prepared as follows: 2–9 μl template DNA, 18 pmol forward primer, 18 pmol reverse primer, 5 pmol probe, 2xSupermix for Probes no UTP (Bio-Rad), and AccuGENE™ Molecular Biology Water (Lonza) to a final volume of 22 μl. Complete lists of applied ddPCR assays are provided in Additional file 9: Tables S3 and S5. One-nanoliter droplets were generated on the QX200 AutoDG Droplet Generator (Bio-Rad). The median number of droplets (partitions) was 16,218 (interquartile range 14,896–17,235). After droplet generation, samples were amplified by PCR in a S1000 Thermal cycler (Bio-Rad) at 95 °C for 10 min and 45 cycles of 95 °C for 30 s, 56 °C for 1 min, and 98 °C for 10 min. Amplified samples were stored at 4 °C for up to 17 h before analysis on the QX200 reader (Bio-Rad). Positive and no-template controls were included for each assay in each plate. Furthermore, for methylation-specific assays a negative control was also included. For methylation-specific assays, the positive and negative controls were 5 ng human methylated and 66 ng non-methylated DNA standards (Zymo Research), respectively. For the CF, Chr3, and PBC assays the positive control was 5 ng human leukocyte DNA. For the CPP1 assay, the positive control was 7000 CPP1 DNA fragments. For fresh frozen tumour tissue and PBL test samples, the DNA input was 5 ng and 66 ng, respectively (quantified prior to bisulfite conversion). Quantasoft v1.7 software (Bio-Rad) with standard settings was used for analysis of ddPCR data from all, but the plasma samples. Plasma samples were analysed using a custom analysis pipeline (see the “Data analysis” section below).

### Methylation-specific droplet digital PCR

Plasma samples were analysed on the Droplet Digital PCR System (Bio-Rad) according to manufacturer’s instructions (Bio-Rad) and performed in accordance with the Minimum Information for Publication of Quantitiative Digital PCR Experiments [30] (dMIQE) guidelines (Additional file 9: Table S6). ddPCR assay information is provided in Additional file 9: Table S3. Each plate included a positive control (5 ng human methylated DNA), a negative control (66 ng human unmethylated DNA), and a no-template control.

### Data analysis

The raw fluorescence intensity data for all individual droplets in each well was extracted using Quantasoft and analysed plate-wise. We used fluorescence data from a fully methylated positive control sample on each plate to identify fluorescence maxima (for the negative and positive droplet populations) and minimum. This was done using a Gaussian kernel density estimator with the smallest possible bandwidth that identified exactly two maxima and one minimum (Additional file 8: Figure S8). All test samples on the plate were subsequently normalized to the median fluorescence of the negative population from the positive control. The fluorescence threshold for calling droplets positive or negative was finally set for all wells, at the minimum point between the negative and positive populations as defined by the positive control sample. The concentration *c* (copies per well) of methylated DNA calculated as *c* = − *N* × ln(1 − *P*/*N*), where *N* is the total number of droplets and *P* is the number of positive droplets [31]. The code in the R language is available at GitHub (https://github.com/MOMA-CRC/ddanalyzor.git).

### Statistical analysis

The predictive accuracy of the individual markers *C9orf50*, *KCNQ5*, and *CLIP4* was estimated by ROC analysis using the R package ROCR. The sensitivity and specificity of the TriMeth test were estimated with corresponding 95% confidence intervals.

## Supplementary information

**Additional file 1:** **Figure S1**. Examples of bisulfite sequencing of the *C9orf50* DNA methylation marker region. a, Colorectal tumour tissue. b, Peripheral blood leucocytes. c, Normal colorectal mucosa. The selected *C9orf50* Infinium HumanMethylation450K BeadchipⓇ array CpG site is marked with green boxes, the location *C9orf50* methylation-specific ddPCR assay primers are probe are marked with black arrows and lines and CpG sites in the assay are marked with black boxes. ddPCR: droplet digital PCR.

**Additional file 2:** **Figure S2.** Biomarker candidate performance in blood and colorectal tumour tissue. Proportion of 27 PBL (a) and 36 CRC tumour tissue (b) samples that were positive for the candidate DNA methylation markers shown on the x-axis. For all markers, samples were scored as positive if they showed any positive signal by ddPCR. *SPG20*, *CDK14*, *EVC*, *KIAA1026*, *UNC5C*, and *ADHFE1* markers were positive in >7.5% of PBL samples and were not tested in CRC tumour tissue. 12 markers that were blank in PBL samples and positive in >93% of CRC tumour tissues were selected for test in plasma. PBL: Peripheral Blood Leucocytes, CRC: Colorectal Cancer, ddPCR: droplet digital PCR.

**Additional file 3:** **Figure S3.** Biomarker candidate performance in plasma. Proportion of 60 plasma samples from controls (a) and CRC patients (b) that was positive for the 12 candidate DNA methylation markers shown on the x-axis. *C9orf50*, *KCNQ5* and *CLIP4* were blank in plasma from healthy individuals and positive in > 70% of plasma samples from CRC patients. CRC: Colorectal Cancer.

**Additional file 4:** **Figure S4.** *C9orf50*, *KCNQ5*, and *CLIP4* assay and Infinium HumanMethylation450K BeadchipⓇ probe positions. Schematic illustration of the localization of index Infinium HumanMethylation450K Beadchip® CpG sites and methylation-specific ddPCR assays related to the presence of CpG sites in the genomic region of *C9orf50* (a), *KCNQ5* (b), and *CLIP4* (c). CRC-specific methylation of the illustrated regions was confirmed by bisulfite sequencing. CRC: Colorectal Cancer. *CRC-specific methylation of CpG sites verified by bisulfite sequencing.

**Additional file 5:** **Figure S5.** Duplex optimization of *C9orf50*, *KCNQ5*, and *CLIP4* DNA methylation markers. Comparison of the performance of *C9orf50* (a), *KCNQ5* (b), and *CLIP4* (c) DNA methylation markers as singleplex and duplex reactions. *C9orf50* and *KCNQ5* was duplexed and *CLIP4* was duplexed with a CF control assay. 3-point 2-fold dilution series of human methylated DNA standard were used as templates and a no template control was included in all reactions. CF: Cytosine-Free.

**Additional file 6:** **Figure S6.** Receiver operating characteristic curves for *C9orf50*, *KCNQ5*, and *CLIP4* in plasma (all UICC stages). Stage-stratified ROC curves from test of *C9orf50*, *KCNQ5* and *CLIP4* individual marker assays in plasma from CRC patients and controls in the test cohort. ROC: Receiver operating characteristic, CRC: Colorectal Cancer.

**Additional file 7:** **Figure S7.** Examples of positive and negative experimental results of ddPCR assays used in plasma. Examples of Quantasoft amplification plots for positive and negative ddPCR results of all assays used in plasma. Samples include from left to right: 2 x CRC patients, 2 x healthy controls, positive control and no template control. The test result (positive or negative) is indicated below each sample. CRC:Colorectal Cancer, ddPCR: droplet digital PCR.

**Additional file 8:** **Figure S8.** Schematic illustration of analysis of ddPCR data from plasma samples. a, A probability density function is estimated from raw fluorescence data of a positive control (#ctrl), which is included on all plates (top panel). Smoothing is done using a gaussian kernel estimator with the smallest bandwidth that results in exactly two maxima and one minimum defining the negative (red) and positive (blue) population of the control sample (bottom panel). Dashed line indicates the median of the negative population. b, Raw fluorescence intensity data (grey) from each sample is normalized to the control sample (#ctrl) so that the medians of the negative populations are similar and equal to that of the control. Shown are #ctrl and 13 samples. Negative and positive populations are finally determined according to the minimum point of the control sample (vertical line). The DNA concentration in each sample is calculated as described in the Methods section.

**Additional file 9:** **Table S1.** DNA methylation level of *C9orf50*, *KCNQ5* and *CLIP4* obtained from Infinium. HumanMethylation450K BeadChip® array data. **Table S2.** Primer sequences used for bisulfite sequencing of candidate biomarker regions. **Table S3.** Overview and primer and probe sequences for methylation-specific ddPCRT of the 29 selected biomarker candidates. **Table S4.** Sensitivities and specificities in plasma from CRC patients and healthy controls (test cohort) for individual markers and the TriMeth test. **Table S5.** Primer and probe sequences for ddPCR control assays. **Table S6.** Digital MIQE checklist for authors, reviewers and editors.

## Data Availability

The datasets used and analysed during the current study are available from the corresponding author on reasonable request.

## Abbreviations

450K: Infinium HumanMethylation450K BeadChip
AML: Acute Myeloid Leukaemia
AUC: Area under the curve
BCL: B-Cell Lymphoma
BS-cfDNA: Bisulfite-converted cfDNA
cfDNA: Circulating cell-free DNA
CRC: Colorectal cancer
ctDNA: Circulating tumour DNA
ddPCR: Droplet digital PCR
FIT: Faecal Immunochemical Test
HNSCC: Head and neck squamous cell carcinoma
PBLs: Peripheral blood leukocytes
ROC: Receiver operating characteristics
UICC: Union for International Cancer Control
