# Supplementary figures and images for: Novel DNA methylation biomarkers show high sensitivity and specificity for blood-based detection of colorectal cancer—a clinical biomarker discovery and validation study
Source: Clin Epigenetics. 2019 Nov 14;11:158. doi: 10.1186/s13148-019-0757-3 (PMC6854894; doi:10.1186/s13148-019-0757-3)

Supplementary Figure 1

a

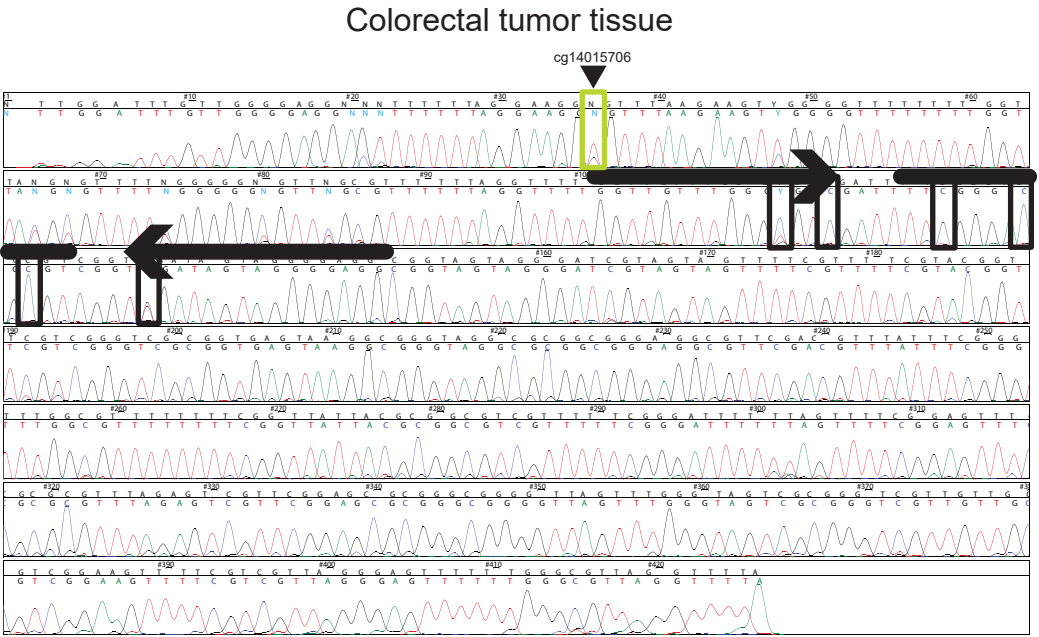

b

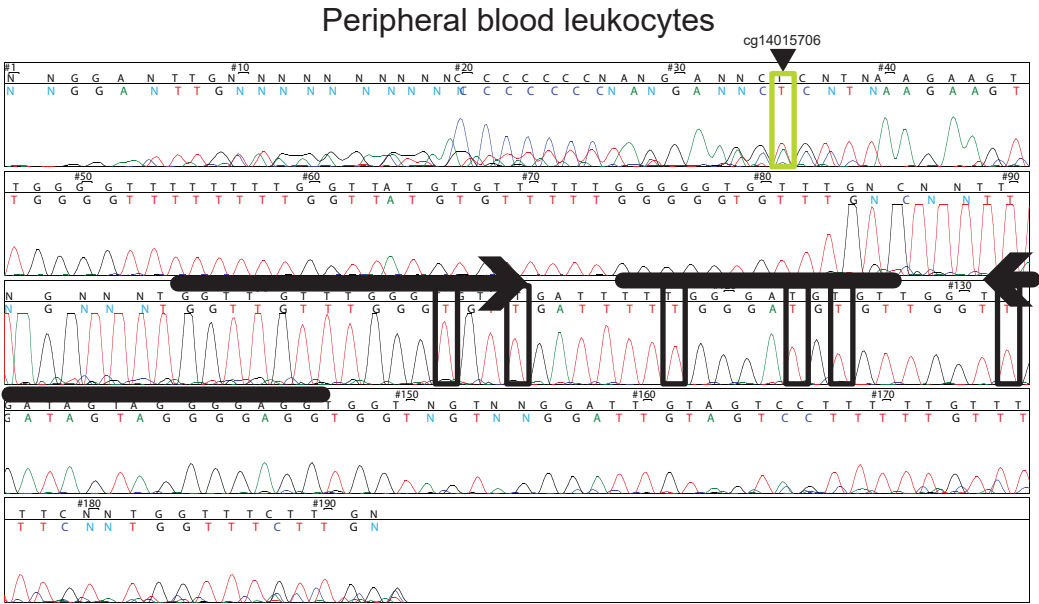

c

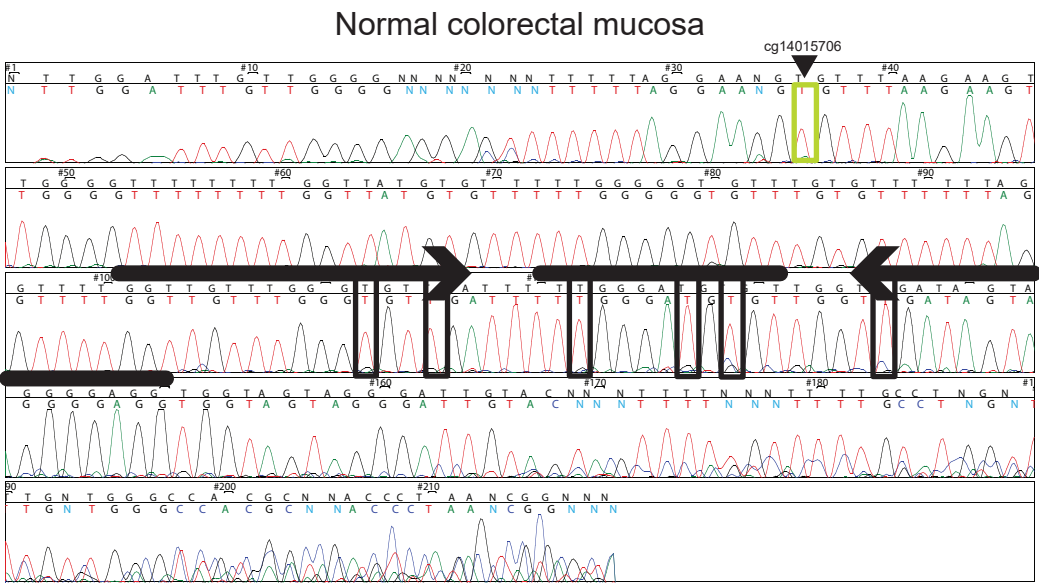

Supplement: Supplementary file 1 — Additional file 1: Figure S1. Examples of bisulfite sequencing of the C9orf50 DNA methylation marker region. a, Colorectal tumour tissue. b, Peripheral blood leucocytes. c, Normal colorectal mucosa. The selected C9orf50 Infinium HumanMethylation450K BeadchipⓇ array CpG site is marked with green boxes, the location C9orf50 methylation-specific ddPCR assay primers are probe are marked with black arrows and lines and CpG sites in the assay are marked with black boxes. ddPCR: droplet digital PCR. [file 13148_2019_757_MOESM1_ESM.pdf]

Supplementary Figure 2

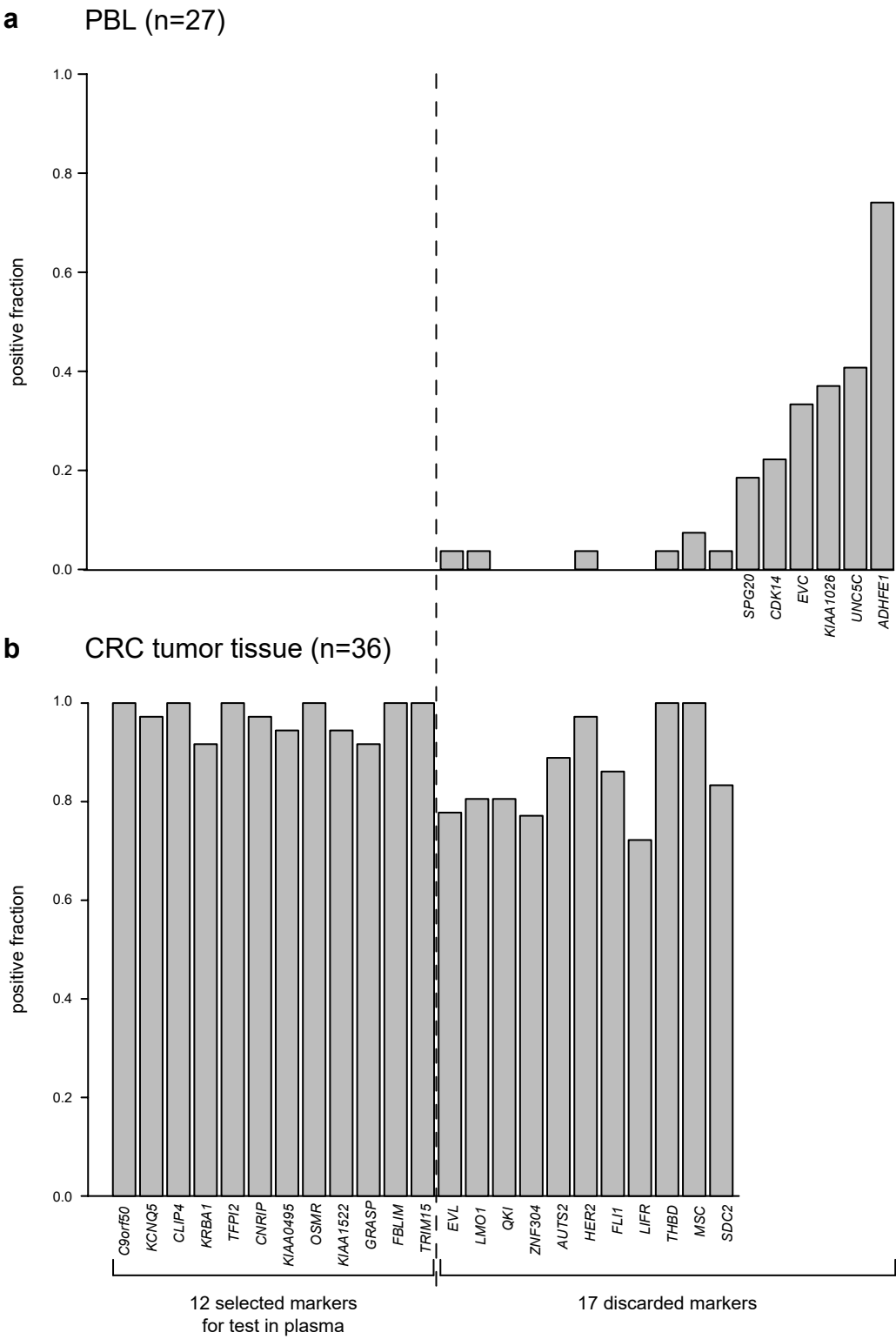

Supplement: Supplementary file 2 — Additional file 2: Figure S2. Biomarker candidate performance in blood and colorectal tumour tissue. Proportion of 27 PBL (a) and 36 CRC tumour tissue (b) samples that were positive for the candidate DNA methylation markers shown on the x-axis. For all markers, samples were scored as positive if they showed any positive signal by ddPCR. SPG20, CDK14, EVC, KIAA1026, UNC5C, and ADHFE1 markers were positive in >7.5% of PBL samples and were not tested in CRC tumour tissue. 12 markers that were blank in PBL samples and positive in >93% of CRC tumour tissues were selected for test in plasma. PBL: Peripheral Blood Leucocytes, CRC: Colorectal Cancer, ddPCR: droplet digital PCR. [file 13148_2019_757_MOESM2_ESM.pdf]

Supplementary Figure 3

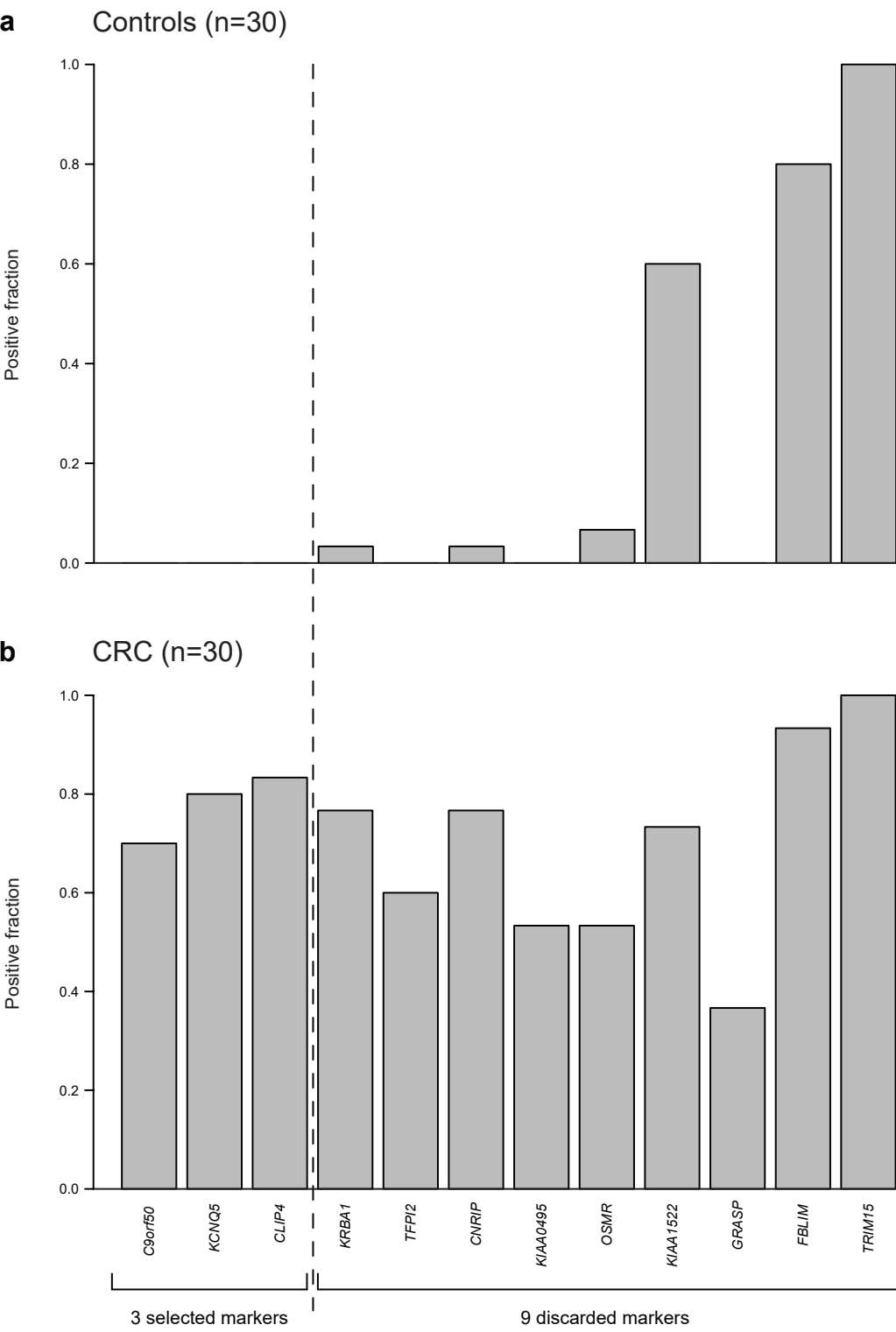

Supplement: Supplementary file 3 — Additional file 3: Figure S3. Biomarker candidate performance in plasma. Proportion of 60 plasma samples from controls (a) and CRC patients (b) that was positive for the 12 candidate DNA methylation markers shown on the x-axis. C9orf50, KCNQ5 and CLIP4 were blank in plasma from healthy individuals and positive in > 70% of plasma samples from CRC patients. CRC: Colorectal Cancer. [file 13148_2019_757_MOESM3_ESM.pdf]

## Supplementary Figure 4

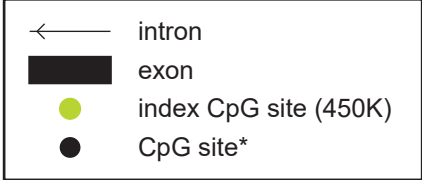

**a** C9orf50

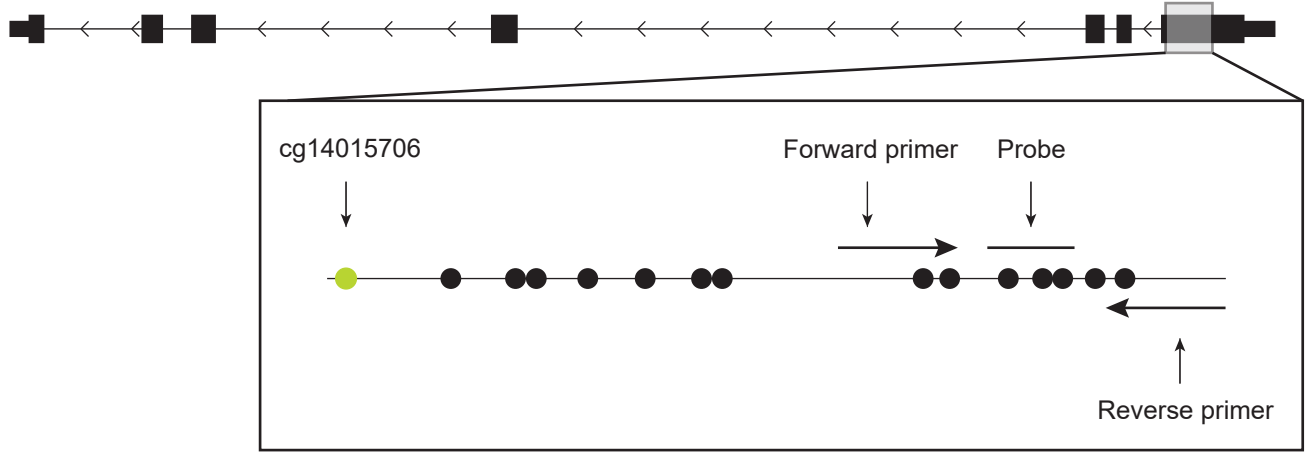

**b** KCNQ5

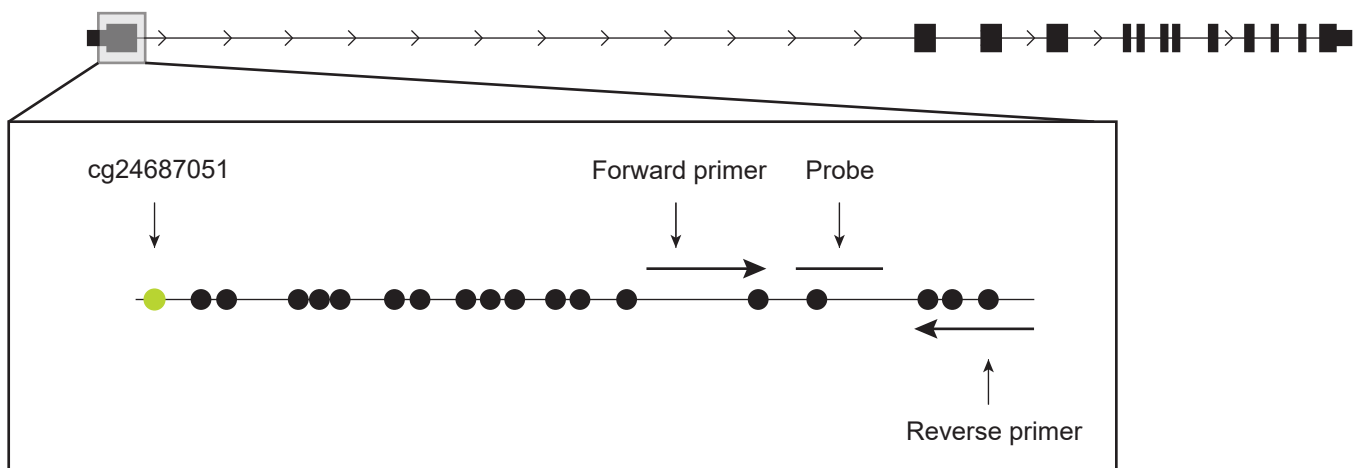

**c** CLIP4

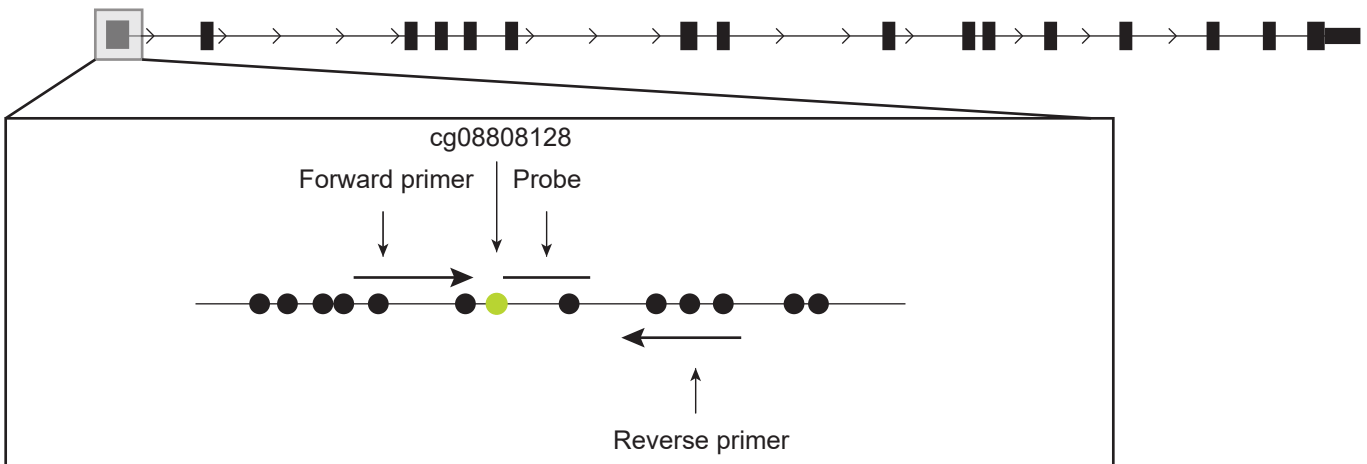

Supplement: Supplementary file 4 — Additional file 4: Figure S4. C9orf50, KCNQ5, and CLIP4 assay and Infinium HumanMethylation450K BeadchipⓇ probe positions. Schematic illustration of the localization of index Infinium HumanMethylation450K Beadchip® CpG sites and methylation-specific ddPCR assays related to the presence of CpG sites in the genomic region of C9orf50 (a), KCNQ5 (b), and CLIP4 (c). CRC-specific methylation of the illustrated regions was confirmed by bisulfite sequencing. CRC: Colorectal Cancer. *CRC-specific methylation of CpG sites verified by bisulfite sequencing. [file 13148_2019_757_MOESM4_ESM.pdf]

Supplementary Figure 5

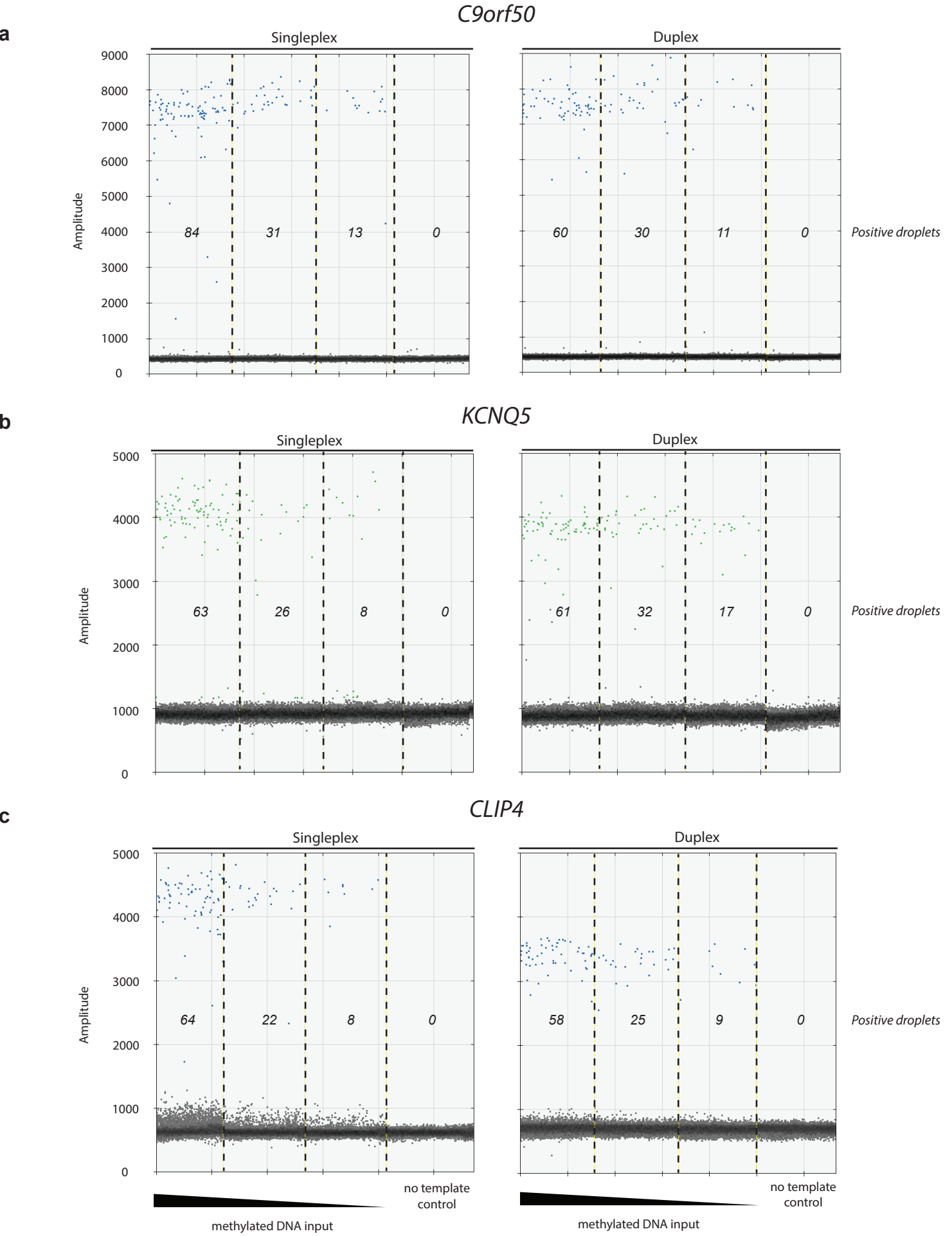

Supplement: Supplementary file 5 — Additional file 5: Figure S5. Duplex optimization of C9orf50, KCNQ5, and CLIP4 DNA methylation markers. Comparison of the performance of C9orf50 (a), KCNQ5 (b), and CLIP4 (c) DNA methylation markers as singleplex and duplex reactions. C9orf50 and KCNQ5 was duplexed and CLIP4 was duplexed with a CF control assay. 3-point 2-fold dilution series of human methylated DNA standard were used as templates and a no template control was included in all reactions. CF: Cytosine-Free. [file 13148_2019_757_MOESM5_ESM.pdf]

Supplementary Figure 6

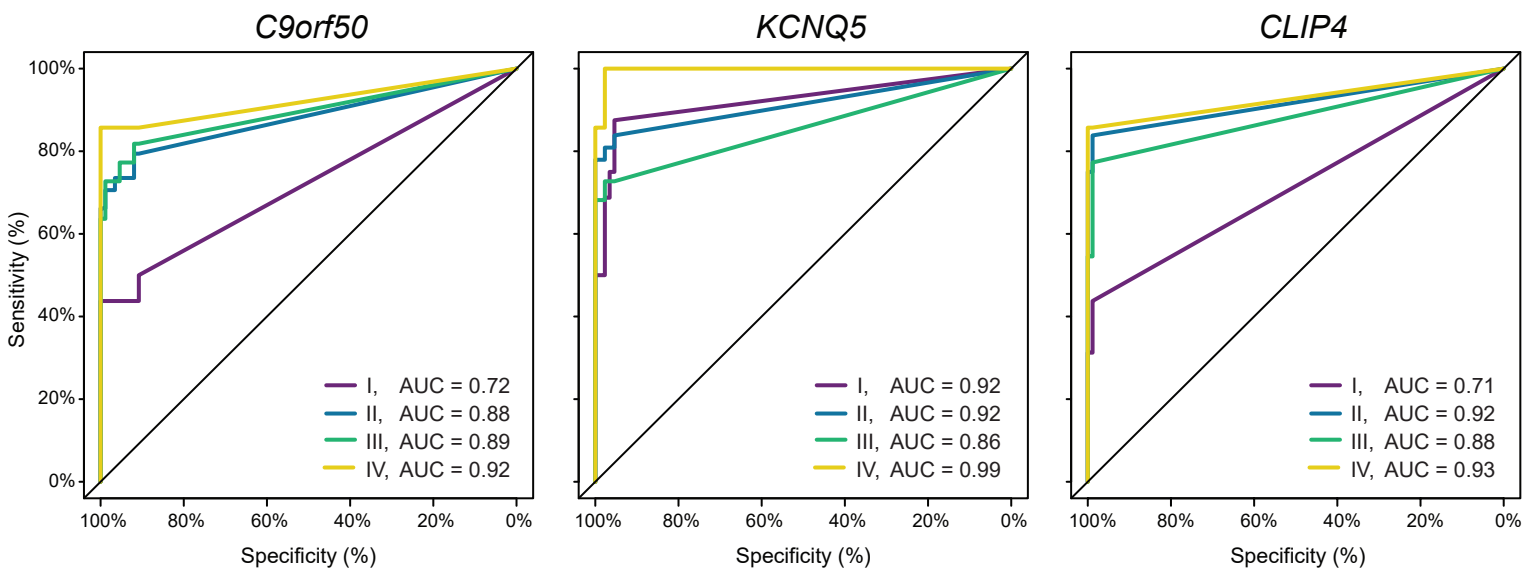

Supplement: Supplementary file 6 — Additional file 6: Figure S6. Receiver operating characteristic curves for C9orf50, KCNQ5, and CLIP4 in plasma (all UICC stages). Stage-stratified ROC curves from test of C9orf50, KCNQ5 and CLIP4 individual marker assays in plasma from CRC patients and controls in the test cohort. ROC: Receiver operating characteristic, CRC: Colorectal Cancer. [file 13148_2019_757_MOESM6_ESM.pdf]

Supplementary Figure 7

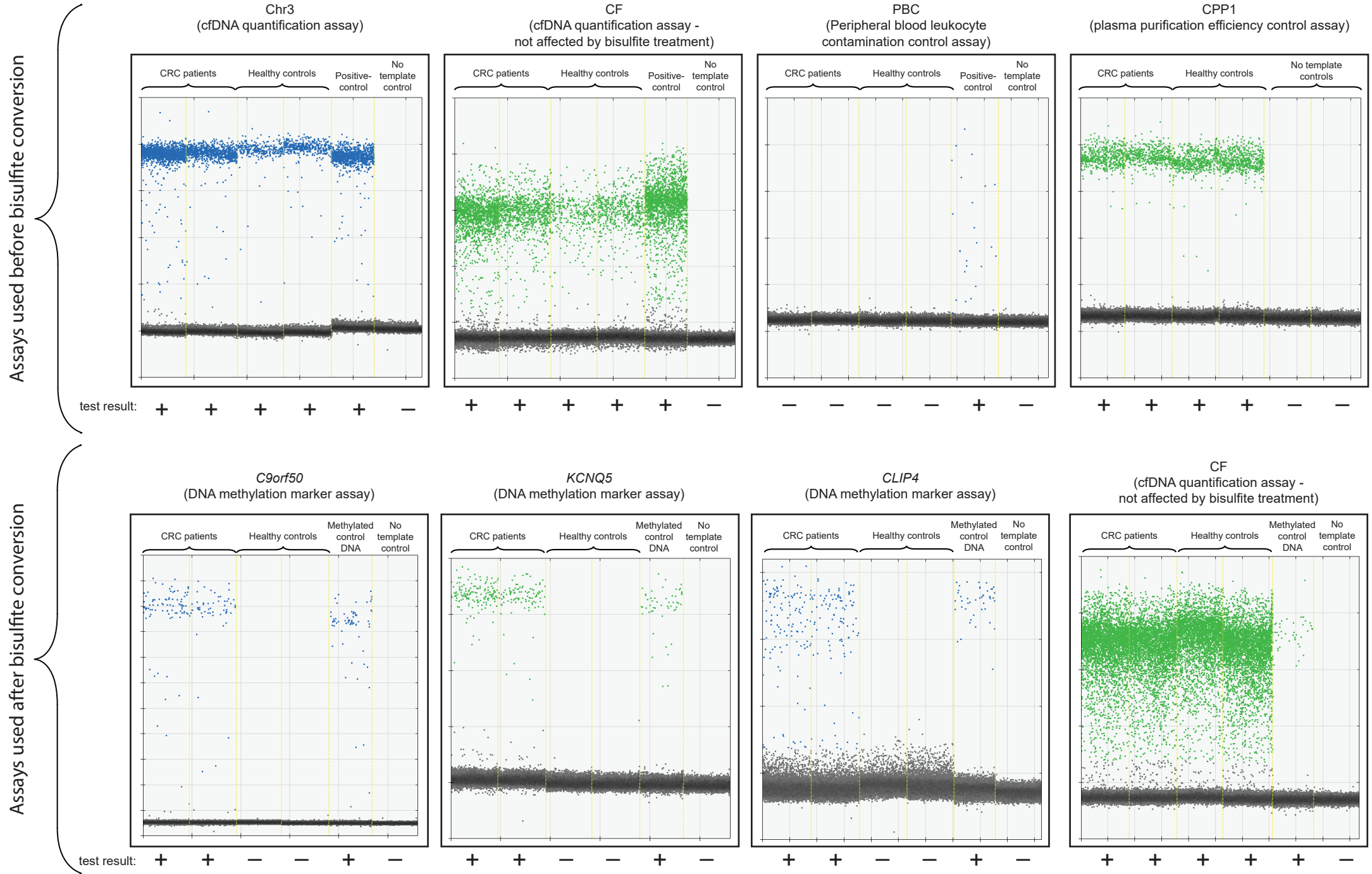

Supplement: Supplementary file 7 — Additional file 7: Figure S7. Examples of positive and negative experimental results of ddPCR assays used in plasma. Examples of Quantasoft amplification plots for positive and negative ddPCR results of all assays used in plasma. Samples include from left to right: 2 x CRC patients, 2 x healthy controls, positive control and no template control. The test result (positive or negative) is indicated below each sample. CRC:Colorectal Cancer, ddPCR: droplet digital PCR. [file 13148_2019_757_MOESM7_ESM.pdf]

Supplementary Figure 8

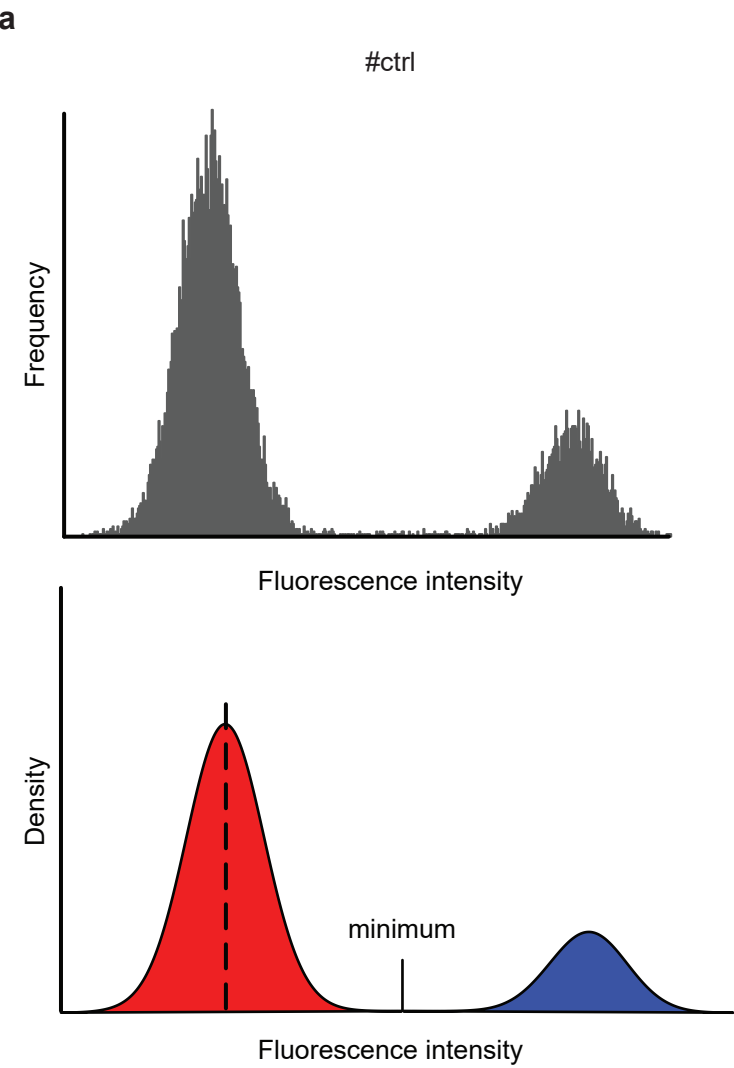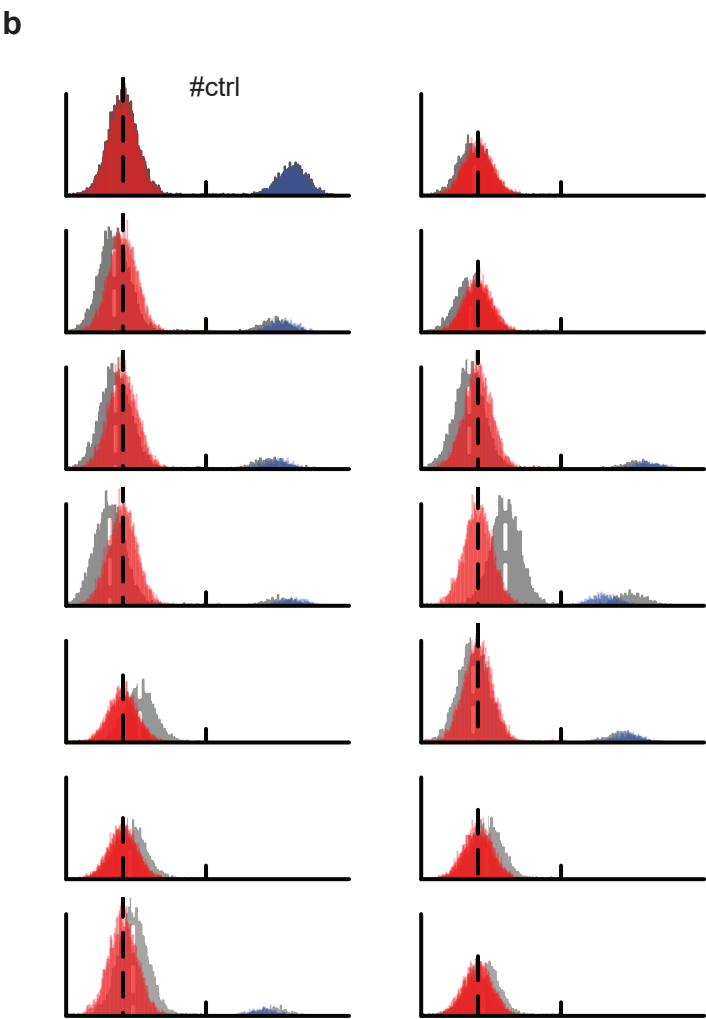

Supplement: Supplementary file 8 — Additional file 8: Figure S8. Schematic illustration of analysis of ddPCR data from plasma samples. a, A probability density function is estimated from raw fluorescence data of a positive control (#ctrl), which is included on all plates (top panel). Smoothing is done using a gaussian kernel estimator with the smallest bandwidth that results in exactly two maxima and one minimum defining the negative (red) and positive (blue) population of the control sample (bottom panel). Dashed line indicates the median of the negative population. b, Raw fluorescence intensity data (grey) from each sample is normalized to the control sample (#ctrl) so that the medians of the negative populations are similar and equal to that of the control. Shown are #ctrl and 13 samples. Negative and positive populations are finally determined according to the minimum point of the control sample (vertical line). The DNA concentration in each sample is calculated as described in the Methods section. [file 13148_2019_757_MOESM8_ESM.pdf]
